# Commentary: IL-21 Receptor Antagonist Inhibits Differentiation of B Cells toward Plasmablasts upon Alloantigen Stimulation

**DOI:** 10.3389/fimmu.2017.00934

**Published:** 2017-08-04

**Authors:** Ashish Kumar Vyas, Nirupma Trehanpati

**Affiliations:** ^1^Department of Molecular and Cellular Medicine, Institute of Liver and Biliary Sciences, New Delhi, India

**Keywords:** B cells differentiation, T follicular helper cells, IL-21R B cell and IL-21

The existence of B cells was discovered in 1890 by Emil von Behring and Shibasaburo Kitasato (1). One of the earliest discovered functions of T cells was to help B cells, which led to the coining of the term “T helper (TH) cell.” The interaction of T cells and B cells was first noted by (2) and further supported by Miller and Mitchell (3).

B cell help by T cells has been elaborately studied since 2000 and resulted in the discovery of interleukin 21 (IL-21). IL-21 is considered a major cytokine, which is required for B cell help. The major sources of IL-21 production are T-follicular helper (TFh) cells and natural killer T (NKT) cells. IL-21 is also the signature cytokine of TFh cells with broad pleiotropic actions including the regulation of B cell differentiation (4). The biological actions of IL-21 have been subject to detailed investigation. We read with great interest the article by de Leur et al. (5), which describes the role of IL-21 in B cell differentiation toward plasmablast (antibody secreting B cells) formation. We do agree with the authors that this study has great relevance in organ transplantation procedures as well as viral or bacterial infections to prevent antigen-driven immune responses. de Leur et al. showed that blockade of IL-21 receptor significantly inhibited B cell differentiation (78%) and subsequent antibody production. However, this blockade did not show any effect on TFh cell frequency or PD-1 or ICOS expression, which is perplexing. These results go against the current understanding that IL-21 is essential for maintenance of TFh cells. IL-21 works in an autocrine manner and is required for maintenance of TFh function through expression of ICOS and PD1. Hence, blockade of IL-21R would be expected to decrease TFh cell differentiation. In this study, the authors have not discussed this aspect, which may be a significant feature of IL-21 functioning in TFh as well as B cell differentiation.

The central role of IL-21 in the germinal center (GC) formation was evident from the direct effects on TFh cell generation (6). T cell help to B cells is a complex mechanism with many factors and signals (stimulatory and inhibitory) involved in this process, especially in the GC. Further studies are required to determine the association of cytokines (IL-21 or IL-6 or IL-4) and their role in different stages of B cell maturation. As B cell differentiation or GC reactions occur in the secondary lymphoid organ, it would be beneficial to conduct this study on secondary lymphoid organs in addition to PBMCs.

Not only does the mechanism of B cell differentiation in circulation depend upon IL-21, it starts when naïve B cells become activated and differentiate into short-lived plasma cells without TFh helpers, which produce low-affinity antibodies. In the GC reaction, B cells interact with TFh cells by direct cell to cell contact or by cytokine secretion and undergo affinity maturation, somatic hyper mutation, and form long-lived memory cells (4). In the GC, memory B cells and plasmablasts are formed with the help of TFh-mediated responses.

According to the current understanding based on recent literature, the differentiation of B cells depends upon the shift from the secretion of IL-21 to IL-4 and not IL-21 alone, as both cytokines together modify the nature of the “help” signals delivered by TFH cells in GC (7). We would suspect that in PBMCs with IL-21R blockade, the status of IL-4R would also be important and needs to be investigated for further understanding the factors affecting B cell differentiation in circulation and GC. Recent studies have shown the role of integrated interactions of other molecules like CD40L, ICOS, CXCR5-CXCL-13, SAP-SLAM, IL-4, and IL-6 in B cell differentiation. However, it is not clear which among these molecules serve as major drivers in normal or different disease conditions. Further studies are required to explore and understand the GC reaction, B cell development, and differentiation in greater detail. The study of the CD40L, ICOS, CXCR5-CXCL-13, SAP-SLAM interaction (8) and gene-regulatory networks (Bach2, BTB, BCL6, BLIMP-1, PAX5, Xbp1, and Prdm1) (9) along with IL-21R function would also be important for identifying major players in B cell differentiation and designing better therapeutics in future.

We congratulate the authors for this remarkable study and recognize that IL-21 is one of the key players in a plasmablast formation and important for B cells as well as TFh differentiation. However, we think the blockade of IL-21R can have both beneficial and detrimental effects. However, the authors suggest that the absence of either IL-21 or IL-6 did not affect TFh differentiation, whereas combined absence of IL-21 and IL-6 led to decreased TFh frequencies. This shows the importance of molecules other than IL-21. For greater insight into B cell differentiation and the role of IL-21, the study of IL-4, IL-6 CD40L, SAP proteins, and regulatory gene networks is also critical since these molecules may play crucial roles in plasmablast formation.

## Author Contributions

AV: conceptual design of study, manuscript writing. NT: editing, critical revision of the article for important intellectual content.

## Conflict of Interest Statement

The authors declare that the research was conducted in the absence of any commercial or financial relationships that could be construed as a potential conflict of interest. The reviewer CH and handling Editor declared their shared affiliation, and the handling Editor states that the process nevertheless met the standards of a fair and objective review.
